# Dengue Fever Knowledge and Awareness Among University Students in Taiz Governorate, Yemen: A Cross‐Sectional Study

**DOI:** 10.1002/hsr2.71112

**Published:** 2025-07-23

**Authors:** Waheed A. M. Ali, Jamil M. A. S. Obaid, Asmaa Alsolihy, Najla M. K. Almekhlafy, Wafa S. Alqadasy, Laila N. Alabsi

**Affiliations:** ^1^ Medical Laboratory Sciences Department, Faculty of Medicine and Health Sciences Al‐Saeed University Taiz Yemen; ^2^ Medical Laboratory Sciences Department, Faculty of Medicine and Health Sciences Taiz University Taiz Yemen; ^3^ Medical Laboratory Sciences Department, Faculty of Medicine and Health Sciences Ibb University Ibb Yemen

**Keywords:** awareness, dengue fever, knowledge, perception, university students

## Abstract

**Background and Aim:**

Dengue fever (DF) is a mosquito‐borne viral disease of great health threat in Yemen. DF is an endemic disease with many outbreaks in Taiz governorate. Among Yemeni communities, university students play an important role in increasing knowledge and good health practices to fight DF. Therefore, this study aimed to measure the DF knowledge and prevention awareness level of this community section.

**Methods:**

This cross‐sectional study was carried out between January and June 2023 among undergraduate students from public and private universities in Taiz City, Yemen. The sample size was 400 students. Participants from different academic levels of different colleges were selected using the stratified random method. Knowledge of dengue fever and prevention awareness was assessed using a validated questionnaire and face‐to‐face interviews. The questionnaire consists of three groups of questions: personal characteristics, general knowledge, and prevention awareness of DF. The question answers were dichotomous: correct and incorrect. Data were analyzed using descriptive statistics and *χ* ^2^ and Odds Ratio (OR) at 95% confidence interval (CI) for association using SPSS 21.

**Results:**

The overall knowledge score of the respondents was 22.2% as good, 68.8% as moderate, and 9% as poor knowledge; meanwhile, that of awareness was 52.8%, 35.7%, and 11.5% respectively. The domain‐specific percentages of correct answers were 57.3%, 61.9%, 83%, 55.3%, and 82.9% for symptoms, causative agent, transmission, clinical management, and prevention awareness. The medical students exhibited higher overall general knowledge and prevention than nonmedical students (OR, 1.912, 95% CI, 1.166–3.134; *p* = 0.013).

**Conclusion:**

Most university students in Taiz exhibited moderate overall knowledge of dengue fever, with strong preventive awareness. Efforts should be made to increase knowledge levels and preventive awareness, specifically among university students, as this will empower them to take proactive measures against dengue fever and promote effective health practices within their communities.

## 1 Introduction

Dengue fever is a viral disease caused by four closely related virus serotypes (DEN‐1, DEN‐2, DEN‐3, and DEN‐4) that can cause DF. These viruses belong to the genus Flavivirus and the Flaviviridae family [1]. In October 2013, the fifth addition (DEN‐5) to the previous four serotypes was announced; this serotype is genetically similar to the other serotypes [2]. Any person living in an endemic area may have an infection with any of these different DV serotypes during their lifetime, as there is no cross‐immunity against other serotypes [3].

Dengue fever is the most common vector‐borne viral infection; it is transmitted by the bite of certain mosquitoes. Two mosquitoes are responsible for transmitting DF in humans: *Aedis aegypti* and *Aedes aldopicus* [4]. DF may appear as a severe, dangerous form, the so‐called dengue hemorrhagic fever, which may lead to severe complications and even death [5]. A global pandemic of DF began in southern Asia during World War II and in the years that followed that conflict [6].

Nowadays, DF represents a public health threat worldwide; more than 100 countries are affected. In 2019 alone, more than 5.2 million cases were reported [7]. This disease is prevalent in countries in tropical and subtropical regions [8]. In Yemen, DF represents a significant public health problem. The first reported and documented DF outbreak occurred in 2002 in the Shapwa governorate, after which other governorates, including Taiz, were distributed [9]. DF in Taiz governorate has become an endemic disease during the last two decades, and outbreaks occur almost annually. In a study conducted during the 2016 DF outbreak in Taiz, 43.3% of a sample of 379 febrile patients who underwent laboratory investigations were proven to have DF [10]. Recent official data for the dengue fever collected from 2020 to 2024 reported an incidence rate of 103.9 per 10,000 and a case fatality rate of 0.21%. The highest proportions of them (39.2%) were reported in Taiz governorate [11].

Despite intensive research on different antiviral strategies, there are no specific antiviral drugs to treat DF [12, 13]. Studies have shown that early detection and proper access to medical care significantly reduce fatality rates. Similarly, mosquito control, which relies heavily on community education and participation, is a critical preventive measure [14, 15]. The improvement of the community members' knowledge, attitudes, and practices (KAPs) can help improve health outcomes as well as disease prevention and control. In Yemeni communities, where a considerable proportion of individuals are illiterates or have low educational levels [16], university graduates and students play a significant role in disseminating knowledge and promoting good health practices during epidemics, alongside other sources of information and guidance. Therefore, assessing university students' KAPs of DF is crucial for the improvement of their knowledge and consequently, the community behavior. It can help health planners to improve their interventions for DF early detection, access to medical care, and implementation of preventive measures.

Lack of KAP studies could represent a barrier to effective dengue control. To our best knowledge, only one study in 2016 had assessed KAP status in urban communities in Taiz governorate [17]. Thus, this study aims to evaluate the university students; KAPs regarding DF to help health authorities in Yemen to formulate more effective treatment, prevention, and control strategies.

## 2 Methods

This conservational cross‐sectional study was carried out between January 2023 and June 2023 to assess overall knowledge regarding DF and awareness of prevention among undergraduate students from governmental and private universities in Taiz city, Yemen. A sample size was calculated manually using Steven Thompson's formula [18].

$$n=\frac{N*p\left(1-p\right)}{\left[N-1*\left(\frac{d^{2}}{z^{2}}\right)\right]+p\left(1-p\right)}$$

*n*: sample size

*N*: population size (17,700)

*z*: 1.96 for CI 95%

*d*: error proportion (0.05)

*p*: probability (0.5)

The formula indicated that the appropriate sample size of 376. For more confidence, the sample size in this study increased to 400 students. The stratification was first built for different universities and divided as a proportion according to their total student numbers. The second stratification was built according to the faculties and the academic level. Finally, the desired number was randomly selected through a lottery of these numbers. This study was approved from the ethical committee of the MLS department “Approval No. 6 MLS Nov, 2022”. Informed oral consent was obtained for each participant, in addition to the prior agreement of their universities.

Data were collected using a structured questionnaire adapted from previous studies [19, 20], validated by four research experts, and examined with 10 participants (it was evaluated with Cronbach's alpha). The questionnaire comprised three sections: demographics (age, gender, university type, faculty, and residence), knowledge of dengue fever, which consists of 18 questions covering symptoms, causative agents, vectors, transmission, and clinical management, and prevention awareness; 12 questions assessing preventive measures and community practices.

### 2.1 Scoring and Categorization [21, 22]

- Domain‐specific analysis: Performance in domains (e.g., symptoms, transmission) was reported as average percentages of correct responses for each domain's questions.
- Total scores: Each correct answer was assigned as 1 and an incorrect as 0. The total score for overall knowledge (from 0 to 18) and that of overall awareness (from 0 to 12) were categorized according to Bloom's cutoff [21] and classified as good, moderate and poor (good: ≥ 80%, moderate: 60%–79%, and poor: < 60%). Thus, the classification according to the total score was > 14, 11–13, and < 10 for overall knowledge, and > 10, 8–9, and < 7 for prevention awareness, respectively. Knowledge was categorized by the summation of correct responses to 18 questions. Awareness was categorized by the summation of correct responses to 12 questions. Bloom's cutoff [21] was applied to total scores but not to individual domains as follows:

### 2.2 Statistical Analysis

Data were analyzed using IBM SPSS 21. Descriptive statistics (frequencies, percentages) summarized participant characteristics and domain‐specific performance. *χ* ^2^ tests examined associations between categorized overall knowledge and awareness level categories (poor, moderate, good). Cross‐tabulation and Odds Ratio were used to analyze the association between overall general KAP and demographic variables. The statistical significance was determined with a *p* value < 0.05.

## 3 Results

The questionnaire was distributed to 400 university students; any student who didn't provide consent was replaced by another. All participants were undergraduate students between the ages of 18 and 26 years.

All 400 participants successfully completed the questionnaire; 63.5% were males, most of the participants were from public universities (70.8%), and the remaining participants (29.2%) were from private universities. A total of 27.8% of the participants were medical, whereas the remaining 72.2% were nonmedical students. Most of the students enrolled in this study (68.5%) lived in Taiz city (an urban area), whereas the remaining (31.5%) were rural residents (Table 1).

**Table 1:** Demographics of the university students included in the study.

Categories		Number	Percentage
Sex	Male	254	63.5
	Female	146	36.5
Type of university	Public	283	70.8
	Private	117	29.2
Study specialty	Medicine or para‐medical sciences	111	27.8
	Nonmedical	289	72.2
Residence	Urban	274	68.5
	Rural	126	31.5

**Figure 1:**
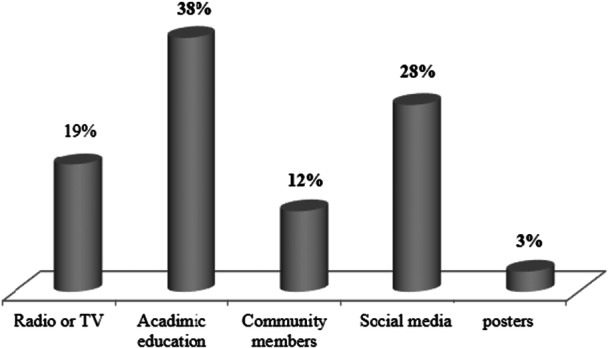
Sources of information of university students on DF.

A total of 396 of 400 university students (99%) who had heard about DF participated in this study. Regarding the sources of information about DF, most of the participants (33%) reported that they had heard about DF through academic education and from social media (28%), followed by radio and/or TV (19%), community members (12%), and posters (3%) as depicted in Figure 1.

Tables 2 and 3 present the frequency and percentage of students' responses (correct and incorrect) to various statements designed to judge their DF knowledge and prevention awareness, respectively. The domain‐specific percentages of the correct answers were as follows: symptoms and complications: 57.3%, causative agent/vector: 61.9%, transmission: 83%, clinical management: 55.5% prevention awareness: 82.9% (Table 4). Regarding the overall knowledge, of the total 400 respondents, 22.2% had good, 68.8% had moderate, and 9% had poor knowledge; meanwhile, the awareness score categorization showed 52.8% had good awareness in comparison with 35.7% who had moderate and 11.5% who had poor awareness, as listed in Table 5.

**Table 2:** Response count of university students with respect to DF knowledge (*n* = 400).

No.	Variables	Correct answer		Incorrect answer	
		Number	%	Number	%
1	Dengue is a viral disease	272	68	128	32
2	Dengue is a serious disease	280	70	120	30
3	Dengue is communicable disease	318	79.5	82	20.5
4	Dengue is transmitted from mother to fetus	266	66.5	134	33.5
5	Dengue may be transmitted through direct contact	373	93.3	27	6.8
6	Dengue may be transmitted through food or drink	355	88.8	45	11.3
7	Flies are possible vectors for DF	389	97.3	11	2.8
8	Primary vector for DF is certain species of mosquitoes	387	96.8	22	5.5
9	The mosquito vectors for DF and malaria are the same	266	66.5	134	33.5
10	Dengue mosquito bites primarily during daytime	104	26	296	7
11	Specific characteristics of *Aedes aegypti* easily by naked eye	202	50.5	198	49.5
12	Dengue mosquito lays eggs in clean water	112	28	288	72
13	Incubation period for DF is about 1 week	229	57.3	171	42.8
14	Known symptoms of DF are fever, headache, and pain in muscles and joints.	298	74.5	102	25.5
15	Hemorrhagic fever is the most dangerous stage of DF	111	27.8	289	72.3
16	No specific medication for DF	132	33	268	67
17	Most cases of DF can be treated at home with pain medicines	280	70	120	30
18	Antibiotics are of benefit in DF	254	63.5	146	36.5

**Table 3:** Response count of university students with respect to DF prevention (*n* = 400).

No.	Variables	Correct answer		Incorrect answer	
		Number	%	Number	%
1	Dengue fever is a preventable disease	348	87	52	13
2	There is a vaccine for DF	238	59.5	162	40.5
3	Controlling of DF vector is an effective method of prevention	322	80.5	78	19.5
4	Community level prevention can reduce DF	360	90	40	10
5	Using preventive methods at home greatly reduces DF incidence	332	83	68	17
5	Removal of open clear stagnant water at house and its surroundings greatly reduce DF vectors and so DF incidence	355	88.8	45	11.3
7	Routine checking of household breeding sites such as water storage tanks, containers in the toilets, and the Kitchen may prevent DF	316	79	84	21
8	Using windows screens and mosquitoes nets effectively prevents DF	332	83	68	17
9	Dengue fever can be prevented by using insecticide	370	92.5	30	7.5
10	Dengue fever can be prevented by using local body mosquito repellent	202	50.5	198	49.5
11	Dengue fever is effectively prevented by fumigation	382	95	18	4.5
12	Wearing protective clothes may prevent DF	280	70	120	30

**Table 4:** Percentage of correct answers for DF knowledge and prevention awareness.

Variables	Percentage of correct answers
Knowledge of symptoms and complications	57.3%
Knowledge of causative agent and vector	61.9%
Knowledge of transmission	83%
Knowledge of clinical management	55.5%
Awareness of dengue prevention and control	82.9%

**Table 5:** Association between students' overall knowledge of DF score categories and prevention awareness score categories.

Variables no. (%)		Overall knowledge			*χ* ^2^	*p*
		Poor 36 (9)	Moderate 275 (68.8)	Good 89 (22.2)		
Prevention awareness	Poor 46 (11.5)	4 (11)	34 (12.4)	8 (9)	14.67	0.005
	Moderate 143 (35.7)	12 (33.3)	84 (30.5)	47 (52.8)		
	Good 211 (52.8)	20 (55.6)	157 (57.1)	34 (38.2)		

Tables 5 and 6 tabulate the results of multivariant analysis of the associations that were carried out via the *χ* ^2^ test to determine the associations between students' overall knowledge and prevention awareness and between students' knowledge and their demographic variants, respectively. There is a statistically significant association between the level of knowledge and prevention awareness (*p *= 0.005), Of the demographic data, the type of faculty (medical and paramedical as a predictor factor) was associated with overall general KAP with an odd of 1.912% and 95% confidence interval of 1.166–3.134, *p* = 0.013 (Table 6).

**Table 6:** Associations between overall general knowledge of DF and student demographic variables.

Variables		Correct answers	OR 95% CI	*p* value
Gender	Male	168	0.957	0.928
	Female	98	0.621–1.474	
University	Public	201	1.179	0.565
	Private	79	0.741–1.876	
Faculty	Medical or paramedical	84	1.912	0.013
	Nonmedical	179	1.166–3.134	
Residence	Urban	177	1.086	0.798
	Rural	79	0.701–1.68	

## 4 Discussion

Mosquito‐borne diseases, including DF, represent important health hazards in many tropical and subtropical areas because of the alarming increase in the number of infected individuals, the burden of the disease, and geographical spread [23]. To adopt health development policies in society, increasing the general and preventive knowledge of health care workers as well as the general population for mobilizing community actions in the development and improvement of the collective and individual health of community members is very important and necessary [24]. Therefore, the assessment of knowledge regarding DF and its vectors is a research priority for understanding the health education and training needs of healthcare workers [25].

The majority of the students enrolled in this study were male (63.5%), whereas only 36.5% were female. This nearly represents the overall percentage of male to female university students in Taiz city, Yemen, due to some traditional habits that are followed by some parents and prohibit girls from higher education.

The present study revealed that 396 of 400 respondents (99%) had heard about DF from media sources (radio, TV, and social media), being the primary means of information dissemination, followed by academic education. This is due to the early time of the first DF epidemic that began about 10 years ago, while they were at school and not enrolled in colleges. Thus, their main sources of information were radio, TV, and social media, not academic education. In studies conducted in Iran, approximately 83.8% of respondents reported awareness of DF [26]. Most respondents in this study had heard about DF because of the high prevalence rate of this disease in Yemen, particularly in Taiz governorate. In regions of high prevalence of disease, it tends to become a prominent topic of discussion in the community, media, and healthcare settings, leading to increased public awareness.

In other studies, conducted in several Asian countries, including India, Indonesia, Myanmar, the Philippines, and Thailand, the vast majority of respondents (> 90%) reported awareness of the disease through media sources [27, 28]. This consistency across diverse geographical regions underscores the critical role of media in disseminating health information and shaping public awareness. Nevertheless, the academic study must be the major source of information for about 38% of participants, higher than other sources.

The results revealed a concerning lack of knowledge regarding dengue fever symptoms, complications, and management (57.3% correct answers), which aligns with findings from studies in Pakistan, Malaysia, and Bangladesh [29, 30, 31]. This gap in awareness could be attributed to several factors: (1) the wide range of clinical manifestations observed in dengue patients, which may lead to confusion with other febrile illnesses such as malaria, influenza, and typhoid [23]; (2) the focus of educational campaigns on transmission, prevention, and control rather than symptoms and complications; and (3) the potential for delayed medical care until severe complications arise [32]. In contrast, participants demonstrated moderate knowledge of the disease's causative agent and vector (61.9% correct answers) and good understanding of transmission (82% correct answers), consistent with findings from Malaysia [26]. These results highlight the need for targeted educational interventions that prioritize improving awareness of symptoms and clinical management, as these are critical for early diagnosis, timely medical care, and effective disease control.

The analysis of the collected data revealed strong preventive awareness (82.9%), consistent with studies in Malaysia and Bangladesh [30, 31]. However, domain‐specific gaps were noticed in knowledge of symptoms (57%) and clinical management (56%), highlighting the need for targeted educational campaigns. These gaps may arise from the focus on vector control in existing public health messaging, overshadowing clinical education [29, 31]. In Yemeni communities, where significant proportions of individuals are illiterate or have low educational levels [16], all of them trust literates generally and university students specifically for information about health problems. So, the role of academic education becomes critical. University graduates and students not only serve as knowledge bearers but also as catalysts for change in health practices. Their advanced training equips them with the skills to disseminate crucial health information and implement effective prevention strategies. This study aimed to measure the knowledge and awareness levels of this community section, underscoring the importance of academic education in enhancing public health literacy and combating diseases like DF.

The strong positive correlation between domain‐specific knowledge and preventive awareness (*p* = 0.005) highlights the importance of integrated education. Medical students showed superior bits of knowledge across all domains (*p* ≤ 0.01); this may be due to curriculum exposure, reinforcing the role of academic training in health literacy.

The results indicated an association between participants' knowledge and their prevention awareness (*p* = 0.005). The associations between participants' knowledge of DF and four different variables were also measured. The results indicated that association exists between knowledge of DF and the type of faculty (either medical or not) (OR, 1.912, 95%CI, 1.166–3.134; *p* = 0.02), whereas it is absent in terms of gender, type of university (public or private), and residence (urban or rural) (*p* > 0.05). The higher percentage of correct answers among medical and paramedical students is logical because of the nature of their studies. Low levels of knowledge and prevention awareness among others may be due to low interest in health culture and due to their commitment to their nonmedical specialties. In addition, Yemen is one of the developing countries that suffer from education system deterioration under the effect of war and poverty.

In endemic countries, educational programs aimed at enhancing public health awareness about dengue DF are vital, particularly among students. While health authorities in the Taiz governorate have initiated several dengue awareness campaigns, there has yet to be comprehensive research assessing the community's awareness levels to guide future educational efforts. This study, due to resource constraints, focused on specific population segments. Despite the participants demonstrating a reasonable understanding of DF, it is crucial to further educate university students through targeted health care programs. Strengthening knowledge and promoting preventive measures can significantly mitigate the risk of DF. Therefore, it is essential for health authorities and nongovernmental organizations to collaborate in developing and implementing effective strategies to combat the spread of dengue fever in the region.

The study limitations include: First, the study focuses solely on university students, potentially overlooking other segments of the population, including high school students, professionals, or community members, who may also play a crucial role in dengue awareness and prevention. Second, the study is confined to Taiz city, which may not reflect knowledge and perceptions in other areas of Yemen, which could have different educational and health contexts.

## 5 Conclusion

The students of universities in Taiz demonstrated strong preventive awareness (82.9%) but critical gaps in symptom recognition (57%) and clinical management (55.5%). This increases the need for interventions addressing these domains, particularly for nonmedical students, who are essential to harness their role as community health advocates. Utilizing digital platforms and social media is still very effective in reaching a broader audience and having a potential impact. Informed individuals are more likely to adopt preventive measures and encourage others to seek medical attention, thereby reducing the incidence of DF and its severe complications. Improving public health literacy can lead to a more resilient population capable of managing health problems and threats effectively.

Future research should focus on prospective studies that assess the effectiveness of educational interventions over time. Additionally, qualitative research could explore the perceptions and attitudes of community members towards dengue prevention and control measures. Identifying barriers to accessing information and healthcare services in rural areas would also provide valuable insights for tailored interventions. By addressing these gaps, future studies can contribute to a more comprehensive understanding of DF dynamics and further enhance community health outcomes in Taiz governorate.

## Author Contributions

**Waheed A.M. Ali:** conceptualization, writing – original draft, supervision, and project administration. **Jamil M.A.S. Obaid:** conceptualization, data curation, writing – review and editing, formal analysis. **Asmaa Alsolihy:** investigation. **Najla M.K. Almekhlafy:** investigation. **Wafa S. Alqadasy:** investigation. **Laila N. Alabsi:** investigation.

## Ethics Statement

This study met the international ethical guidelines, mainly the WMA Declaration of Helsinki—Ethical Principles for Medical Research Involving Human Subjects, 2013. Ethical approval of ethical committee of MLS departments was No. 6 MLS Nov, 2022.

## Consent

Informed consent was obtained from participants.

## Conflicts of Interest

The authors declare no conflicts of interest.

## Transparency Statement

The lead author, Jamil M.A.S. Obaid, affirms that this manuscript is an honest, accurate, and transparent account of the study being reported; that no important aspects of the study have been omitted; and that any discrepancies from the study as planned (and, if relevant, registered) have been explained.

## Data Availability

All data are available in this manuscript.

## References

[1] A. Murugesan and M. Manoharan , “Dengue Virus.” in Emerging and Reemerging Viral Pathogens: Vol 1: Fundamental and Basic Virology Aspects of Human, Animal and Plant Pathogens, eds. M. M. Ennaji (Academic Press, 2019), 296–374.

[2] M. S. Mustafa , V. Rasotgi , S. Jain , and V. Gupta , “Discovery of Fifth Serotype of Dengue Virus (DENV‐5): A New Public Health Dilemma in Dengue Control,” Medical Journal, Armed Forces India 71, no. 1 (2015): 67–70. 25609867 10.1016/j.mjafi.2014.09.011 PMC4297835

[3] J. L. Deen , E. Harris , B. Wills , et al., “The WHO Dengue Classification and Case Definitions: Time for a Reassessment,” Lancet 368, no. 9530 (2006): 170–173. 16829301 10.1016/S0140-6736(06)69006-5

[4] V. H. Ferreira‐de‐Lima and T. N. Lima‐Camara , “Natural Vertical Transmission of Dengue Virus in *Aedes aegypti* and *Aedes albopictus*: A Systematic Review,” Parasites & Vectors 11, no. 1 (2018): 77. 29391071 10.1186/s13071-018-2643-9 PMC5793400

[5] A. M. Yusuf and N. A. Ibrahim , “Knowledge, Attitude and Practice Toward Dengue Fever Prevention and Associated Factors Among Public Health Sector Health‐Care Professionals in Dire Dawa, Eastern Ethiopia,” Risk Management and Healthcare Policy 12 (2019): 91–104. 31239796 10.2147/RMHP.S195214 PMC6560185

[6] D. J. Gubler , “Dengue and Dengue Hemorrhagic Fever: Its History and Resurgence as a Global Public Health Problem.” in Dengue and Dengue Hemorrhagic Fever, eds. D. J. Gubler and G. Kuno (CAB International, 1997), 1–22.

[7] “World Health Organization (WHO), Dengue and Severe Dengue,” published 2024, https://www.who.int/news-room/fact-sheets/detail/dengue-and-severe-dengue.

[8] W. H. Elson , E. Ortega , M. Kreutzberg‐Martinez , et al., “Cross‐Sectional Study of Dengue‐Related Knowledge, Attitudes and Practices in Villa El Salvador, Lima, Peru,” BMJ Open 10, no. 10 (2020): e03. 10.1136/bmjopen-2020-037408 PMC7539572 33028551

[9] A.‐G. Ma , “Epidemiological Review of Dengue Fever in Yemen,” International Journal of Advanced Research 3, no. 7 (2015): 1578–1584.

[10] K. A. Alghazali , B. T. Teoh , S. S. Sam , et al., “Dengue Fever Among Febrile Patients in Taiz City, Yemen During the 2016 War: Clinical Manifestations, Risk Factors, and Patients Knowledge, Attitudes, and Practices Toward the Disease,” One Health 3, no. 9 (2019): 100119. 10.1016/j.onehlt.2019.100119 PMC7184203 32368608

[11] W. H. Edrees , W. A. Al‐Shehari , A. M. Al‐Haddad , L. M. Alrahabi , O. S. Al‐Haddad , and A. A. Al‐Halani , “Dengue Fever in Yemen: A Five‐Year Review, 2020–2024,” BMC Infectious Diseases 25, no. 1 (2025): 28. 39762726 10.1186/s12879-024-10429-6 PMC11702136

[12] P. Padmanabhan , S. Khaleefathullah , K. Kaveri , et al., “Antiviral Activity of Thiosemicarbazones Derived From α‐Amino Acids Against Dengue Virus,” Journal of Medical Virology 89, no. 3 (2017): 546–552. 27490721 10.1002/jmv.24655

[13] J. Pu , L. He , H. Xie , et al., “Antiviral Activity of Carbenoxolone Disodium Against Dengue Virus Infection,” Journal of Medical Virology 89, no. 4 (2017): 571–581. 27155198 10.1002/jmv.24571 PMC7167157

[14] R. Bellini , H. Zeller , and W. Van Bortel , “A Review of the Vector Management Methods to Prevent and Control Outbreaks of West Nile Virus Infection and the Challenge for Europe,” Parasites & Vectors 7 (2014): 323. 25015004 10.1186/1756-3305-7-323 PMC4230500

[15] H. López‐Gatell , C. M. Alpuche‐Aranda , J. I. Santos‐Preciado , and M. Hernández‐Ávila , “Dengue Vaccine: Local Decisions, Global Consequences,” Bulletin of the World Health Organization 94, no. 11 (2016): 850–855. 27821888 10.2471/BLT.15.168765 PMC5096346

[16] 9th Alexo Observatory Statistical Bulletin, Illiteracy in Arabic Region: The Current Situation Future Estimates by 2030, published March 2023, https://observatory.alecso.org/Data/wp-content/uploads/2023/07/Bulletin%209%20(Ang).pdf.

[17] T. A. A. Alyousefi , R. Abdul‐Ghani , M. A. K. Mahdy , et al., “A Household‐Based Survey of Knowledge, Attitudes and Practices Towards Dengue Fever Among Local Urban Communities in Taiz Governorate, Yemen,” BMC Infectious Diseases 16, no. 1 (2016): 543. 27717333 10.1186/s12879-016-1895-2 PMC5054547

[18] S. K. Thompson , Sampling, 3rd ed. (John Wiley & Sons Inc., 2012).

[19] A. Mohammed Abdelfatah Alhoot , B. Mohammed Faez Baobaid , A. Anis rageh al‐Maleki , et al., “Knowledge, Attitude, and Practice Towards Dengue Fever Among Patients in Hospital Taiping,” Malaysian Journal of Public Health Medicine 17, no. 3 (2017): 66–75.

[20] G. Kurdi , J. Leo , B. Parsia , U. Sattler , and S. Al‐Emari , “A Systematic Review of Automatic Question Generation for Educational Purposes,” International Journal of Artificial Intelligence in Education 30 (2020): 121–204.

[21] L. Udayanga , N. Gunathilaka , M. C. M. Iqbal , K. Pahalagedara , U. S. Amarasinghe , and W. Abeyewickreme , “Socio‐Economic, Knowledge Attitude Practices (KAP), Household Related and Demographic Based Appearance of Non‐Dengue Infected Individuals in High Dengue Risk Areas of Kandy District, Sri Lanka,” BMC Infectious Diseases 18 (2018): 88. 29466952 10.1186/s12879-018-2995-y PMC5822474

[22] M. A. Seid and M. S. Hussen , “Knowledge and Attitude Towards Antimicrobial Resistance Among Final Year Undergraduate Paramedical Students at University of Gondar, Ethiopia,” BMC Infectious Diseases 18 (2018): 312. 29980174 10.1186/s12879-018-3199-1 PMC6035414

[23] P. Nadeeka , G. Padhn , and L. Amarasinghe , “Geographic, Economic and Sociocultural Factors Which Defining the Risk of Dengue Transmission in Kelaniya, Sri Lanka,” Journal of Experimental Biology and Agricultural Sciences 3, no.2 (2014): 158–164.

[24] B. H. B. Van Benthem , N. Khantikul , K. Panart , P. J. Kessels , P. Somboon , and L. Oskam , “Knowledge and Use of Prevention Measures Related to Dengue in Northern Thailand,” Tropical Medicine & International Health 7, no. 11 (2002): 993–1000. 12390606 10.1046/j.1365-3156.2002.00950.x

[25] R. Shirzad , A. A. Alesheikh , M. Ahmadkhani , and S. R. Naddaf , “ *Aedes albopictus*: A Spatial Risk Mapping of the Mosquito Using Geographic Information System in Iran,” Applied Geomatics 13 (2021): 691–700.

[26] S. H. Nikookar , M. Moosazadeh , M. Fazeli‐Dinan , M. Zaim , M. M. Sedaghat , and A. Enayati , “Knowledge, Attitude, and Practice of Healthcare Workers Regarding Dengue Fever in Mazandaran Province, Northern Iran,” Frontiers in Public Health 11 (2023): 1129056. 37469697 10.3389/fpubh.2023.1129056 PMC10352843

[27] S. Harish , S. Srinivasa , P. Shruthi , et al., “Knowledge, Attitude and Practice Regarding Dengue Infection Among Parents of Children Hospitalized for Dengue Fever,” Current Pediatric Research 22, no. 1 (2018): 33–37.

[28] S. Mohapatra and A. Aslami , “Knowledge, Attitude and Practice Regarding Dengue Fever Among General Patients of a Rural Tertiary‐Care Hospital in Sasaram, Bihar,” International Journal of Community Medicine and Public Health 3, no. 2 (2016): 586–591.

[29] A. Abbasi , “Dengue Fever: A Statistical Analysis Regarding Awareness about Dengue Among University Students in Azad Kashmir,” Journal of Healthcare Communications 2, no. 01 (2017): 1–8.

[30] W. R. Wan Rosli , S. Abdul Rahman , J. K. Parhar , and M. I. Suhaimi , “Positive Impact of Educational Intervention on Knowledge, Attitude, and Practice Towards Dengue Among University Students in Malaysia,” Journal of Public Health 27, no. 4 (2019): 461–471.

[31] M. I. Hossain , N. E. Alam , S. Akter , et al., “Knowledge, Awareness and Preventive Practices of Dengue Outbreak in Bangladesh: A Countrywide Study,” PLoS One 16, no. 6 (2021): e0252852. 34111157 10.1371/journal.pone.0252852 PMC8192001

[32] Y. Krishnamoorthy , D. Chandar , V. Jayaseelan , K. Vijayakumar , K. Sivaranjini , and M. Vijayageetha , “Household Survey on Public Awareness and Attitudes Toward Dengue Infection in Rural Tamil Nadu, South India,” Journal of Education and Health Promotion 7 (2018): 171. 30693307 10.4103/jehp.jehp_81_18 PMC6332651

